# Spontaneous Bacterial Peritonitis as a Rare Complication of a Pancreatic Duct Leak in a Patient Without Cirrhosis: A Case Report

**DOI:** 10.7759/cureus.115739

**Published:** 2026-09-03

**Authors:** Rana Afram, John Bajouka, Fred Ahmadi, Pooja J Mude, Edward Yousif

**Affiliations:** 1 Internal Medicine, Henry Ford Health, Southfield, USA; 2 Internal Medicine, Ascension Providence Hospital, Southfield, USA; 3 Internal Medicine, Medical College of Georgia, Augusta University, Augusta, USA; 4 Gastroenterology, Henry Ford Health, Southfield, USA

**Keywords:** endoscopy ercp, non-cirrhotic ascites, pancreatic ascites, pancreatic duct leak, pancreatitis chronic, portal vein thrombosis, spontaneous bacterial peritonitis (sbp)

## Abstract

Spontaneous bacterial peritonitis (SBP) is classically associated with liver cirrhosis and portal hypertension. Its occurrence in non-cirrhotic patients is rare and poorly characterized. Pancreatic ascites, resulting from disruption of the pancreatic duct (PD) or leakage from a pseudocyst, is an unusual cause of ascites in chronic pancreatitis. The enzyme-rich, exudative nature of this fluid can itself cause chemical peritonitis with neutrophilia, mimicking infection.

We report the case of a 50-year-old man with alcohol use disorder and chronic calcific pancreatitis who presented with recurrent massive ascites in the absence of hepatic cirrhosis. Paracentesis revealed a markedly elevated ascitic fluid amylase level (14,619 U/L) and a polymorphonuclear leukocyte (PMN) count exceeding 250 cells/mm³; the ascitic fluid culture was negative. Given the negative culture, we classified the condition as culture-negative neutrocytic ascites (CNNA), with a clinical syndrome highly consistent with SBP, rather than as culture-confirmed SBP. The serum-ascites albumin gradient (SAAG) was 1.5 g/dL, supporting the presence of portal hypertension, which was attributed to nonocclusive portal vein thrombosis (PVT). Imaging identified a PD stricture with upstream dilatation and a leak tracking into the peritoneal cavity. Endoscopic retrograde cholangiopancreatography (ERCP), attempted for therapeutic PD drainage, was unsuccessful because of pancreatic calcifications and distorted ductal anatomy. The admission was further complicated by acute aortoiliac thromboembolism, compartment syndrome, and chylous ascites, all of which were successfully managed with surgical and supportive care. Conservative management with intravenous antibiotics, total parenteral nutrition (TPN), a low-fat diet, and octreotide led to spontaneous resolution of the PD leak, as confirmed by secretin-enhanced magnetic resonance cholangiopancreatography (MRCP).

Neutrocytic ascites clinically consistent with SBP may occur in non-cirrhotic patients when a PD leak and localized portal hypertension coexist. However, the anatomical connection between the PD and the gastrointestinal lumen, together with the limited sensitivity of cultures in this setting, means that such cases may be more accurately classified as CNNA. Clinicians should maintain a high index of suspicion for infectious peritoneal complications in patients with pancreatic ascites while using precise terminology that reflects genuine diagnostic uncertainty.

## Introduction

Spontaneous bacterial peritonitis (SBP) is defined as an infection of ascitic fluid in the absence of an evident, surgically treatable intra-abdominal source and is diagnosed by an ascitic fluid polymorphonuclear (PMN) leukocyte count of ≥250 cells/mm³ [1]. It is one of the most feared complications of advanced liver disease. The overwhelming majority of SBP cases occur in patients with cirrhosis and portal hypertension, in whom altered intestinal permeability, impaired hepatic reticuloendothelial function, reduced ascitic fluid complement activity, and bacterial overgrowth combine to permit bacterial translocation from the gut lumen into the ascitic fluid [2,3]. The reported frequency of SBP among cirrhotic patients with ascites ranges from 10% to 30%, with a historical in-hospital mortality rate of 20-40% [4].

A key pathophysiological distinction underlies this case. Cirrhotic ascites is classically a portal hypertension-related, relatively low-protein fluid, whereas pancreatic ascites is a protein- and enzyme-rich fluid resulting from ductal disruption. This distinction is diagnostically important: pancreatic enzymes leaking into the peritoneal cavity can independently recruit neutrophils and elevate the ascitic PMN count through chemical irritation, even in the absence of bacterial infection. This creates a genuine diagnostic dilemma: the ≥250 cells/mm³ PMN threshold used as a diagnostic criterion for SBP was validated primarily in cirrhotic ascites, and its interpretation in pancreatic ascites is far less certain. Distinguishing a bacterially infected process from sterile chemical peritonitis in this setting is a central theme of the present report.

SBP in non-cirrhotic patients is rare and less well characterized. Reported settings for ascites-associated peritonitis in non-cirrhotic patients include nephrotic syndrome, malignancy, cardiac failure, tuberculosis, and pancreatic disease; in these settings, secondary causes of peritonitis must be carefully considered and may be more common than true SBP [5]. In pancreatic disease specifically, peritoneal fluid infection is typically attributed to infected pseudocysts, pancreatic abscesses, or direct transmural contamination rather than to a translocation-mediated process resembling cirrhotic SBP.

Pancreatic ascites results from the leakage of pancreatic secretions into the peritoneum, most often because of disruption of the main pancreatic duct (PD) or leakage from a pseudocyst. It is an uncommon complication of chronic and acute pancreatitis and occurs most frequently in the setting of alcohol-related pancreatitis [6]. When this chemical peritoneal environment is complicated by concurrent portal vein thrombosis, a recognized vascular complication of chronic pancreatitis and peripancreatic inflammation, portal hypertension and altered gut hemodynamics may theoretically create conditions permissive to bacterial translocation, even without hepatic parenchymal disease [7].

The combination of neutrocytic pancreatic ascites, a documented PD leak, and portal vein thrombosis in a non-cirrhotic patient has, to our knowledge, not been comprehensively described [8,9]. The differential diagnosis in such cases, spanning an infected pseudocyst, secondary bacterial peritonitis, sterile chemical peritonitis, and culture-negative neutrocytic ascites (CNNA), is rarely discussed explicitly. This report presents a case in which SBP, specifically CNNA, was considered, evaluated against this differential diagnosis, and treated, with the goal of clarifying the diagnostic reasoning involved and broadening clinicians’ awareness of this uncommon and clinically demanding scenario.

## Case presentation

A 50-year-old man with a history of alcohol use disorder, chronic calcific pancreatitis with pancreatic pseudocysts, and hypertension presented with a one-month history of progressive abdominal distention and discomfort, as well as an unintentional 10-pound weight loss over 10 months. He reported daily heavy alcohol consumption for many years, with recent attempts to reduce his intake, a 10-pack-year smoking history, and occasional marijuana use. He had no prior history of liver disease, jaundice, variceal bleeding, or hepatic encephalopathy.

Initial contrast-enhanced CT of the abdomen and pelvis demonstrated chronic pancreatitis with superimposed acute inflammatory changes; pancreatic pseudocysts, including a lesion in the head/uncinate process compressing the superior mesenteric vein (SMV); a nonocclusive thrombus in the main and right portal veins; diffuse small-bowel wall thickening suggestive of portal enteropathy; and moderate-to-large-volume ascites (Figure 1).

**Figure 1:**
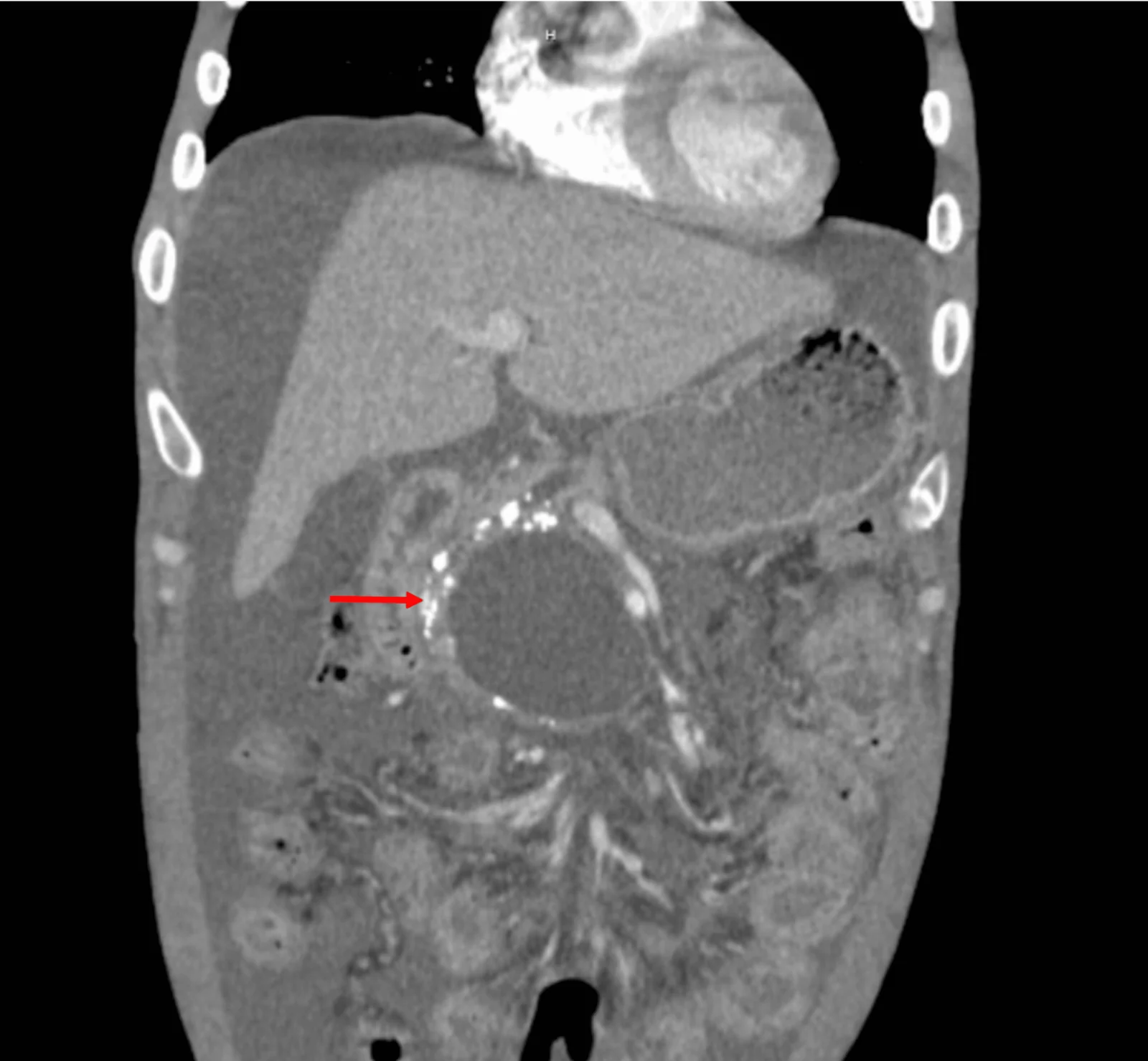
Coronal contrast-enhanced CT image of the abdomen demonstrating a large pancreatic pseudocyst centered in the uncinate process (red arrow), with associated pancreatic calcifications consistent with chronic calcific pancreatitis.

Diagnostic and therapeutic paracentesis, during which 2.2 L of fluid was removed, revealed a WBC count of 1,691 cells/mm³ with 66% PMN leukocytes (absolute PMN count, approximately 1,116 cells/mm³; diagnostic threshold, ≥250 cells/mm³), a markedly elevated amylase level of 14,619 U/L strongly supporting a pancreatic origin, and a SAAG of 1.5 g/dL consistent with portal hypertension. Ascitic fluid glucose, lactate dehydrogenase, and total protein levels were not obtained during this initial paracentesis, precluding the formal application of Runyon’s criteria to assess for secondary bacterial peritonitis; this is addressed further in the Limitations section. Ascitic fluid was sent for culture using standard laboratory processing rather than bedside inoculation into blood culture bottles, and no organisms were recovered. Given the elevated PMN count, negative culture, and inability to fully assess for secondary peritonitis using Runyon’s criteria, the ascitic fluid findings were provisionally classified as CNNA, with a clinical picture highly consistent with SBP. The patient was empirically started on IV ceftriaxone, with a planned transition to oral ciprofloxacin. A repeat diagnostic paracentesis to confirm PMN clearance after antibiotic initiation was not performed; clinical improvement was instead assessed through symptoms, drain output, and serial imaging.

Serum studies were notable for a hemoglobin level of 12.4 g/dL, mean corpuscular volume (MCV) of 102 fL, platelet count of 629 × 10³/µL, sodium level of 132 mmol/L, creatinine level of 0.89 mg/dL, total bilirubin level of 0.3 mg/dL, alanine aminotransferase (ALT) level of 5 IU/L, aspartate aminotransferase (AST) level of 15 IU/L, alkaline phosphatase level of 48 IU/L, and international normalized ratio (INR) of 1.10 (Table 1). Normal transaminase, bilirubin, and coagulation results, together with the absence of clinical stigmata of chronic liver disease, made cirrhosis an unlikely cause of the ascites.

**Table 1:** Diagnostic laboratory and ascitic fluid values. Serum hematologic, metabolic, and hepatic laboratory values obtained at initial presentation, alongside ascitic fluid findings from diagnostic paracentesis performed before antibiotic initiation. ALT: Alanine aminotransferase; AST: Aspartate aminotransferase; CNNA: Culture-negative neutrocytic ascites; INR: International normalized ratio; MCV: Mean corpuscular volume; PMN: Polymorphonuclear leukocyte; SAAG: Serum-ascites albumin gradient; SBP: Spontaneous bacterial peritonitis.

Lab	Inpatient Lab Values	Normal Reference Values
Ascitic Fluid WBC	1,691 cells/mm³	<500 cells/mm³
Ascitic Fluid PMN (%)	66%	<25%
Ascitic Fluid Absolute PMN Count	≈1,116 cells/mm³	<250 cells/mm³ (SBP threshold)
Ascitic Fluid Amylase	14,619 U/L	~30-110 U/L (approx. serum level)
Serum-Ascites Albumin Gradient (SAAG)	1.5 g/dL	<1.1 g/dL (≥1.1 suggests portal hypertension)
Hemoglobin	12.4 g/dL	13.5-17.5 g/dL (male)
MCV	102 fL	80-100 fL
Platelet Count	629 × 10³/µL	150-450 × 10³/µL
Sodium	132 mmol/L	135-145 mmol/L
Creatinine	0.89 mg/dL	0.7-1.3 mg/dL (male)
Total Bilirubin	0.3 mg/dL	0.1-1.2 mg/dL
ALT	5 IU/L	7-56 IU/L
AST	15 IU/L	10-40 IU/L
Alkaline Phosphatase	48 IU/L	44-147 IU/L
INR	1.1	0.8-1.1

MRCP identified a short-segment inflammatory stricture of the PD in the pancreatic head, with upstream ductal dilatation and mild side-branch dilatation in the body and tail; multiple peripancreatic fluid collections, including a dominant uncinate pseudocyst; moderate-to-large-volume ascites; and a long-segment common bile duct stricture attributed to pancreatitis-related inflammation. A definitive site of ductal leakage into the peritoneal cavity was not identified on this initial study, although the overall constellation of findings strongly suggested a PD leak (Figure 2).

**Figure 2:**
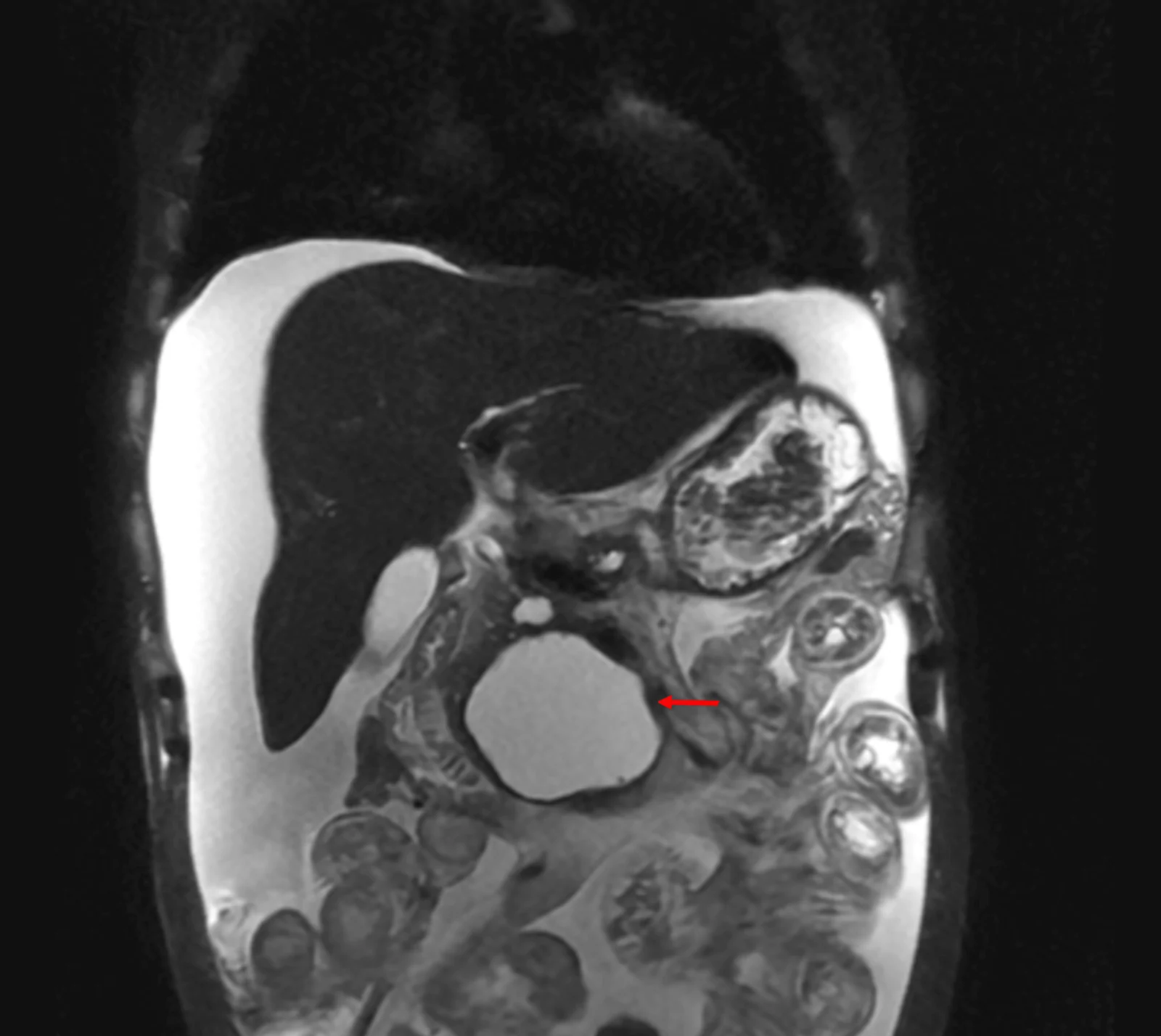
Coronal T2-weighted MRI of the abdomen demonstrating sequelae of chronic pancreatitis. A well-defined, T2-hyperintense pancreatic pseudocyst is identified in the region of the pancreatic head and uncinate process (red arrow), appearing as a homogeneous fluid-signal structure.

Repeat CT demonstrated interval reaccumulation of moderate-volume ascites, diffuse peripancreatic and mesenteric edema, and persistent main PD dilatation. A tract between the duct and the retroperitoneum was described as “difficult to identify, though likely present given the history.” The dominant pseudocyst had increased in size, with persistent SMV narrowing.

CT of the chest, abdomen, and pelvis one week later confirmed extensive pancreatic calcifications; a 6.4 × 6.0 × 6.3 cm head/uncinate pseudocyst; an additional 3.6 × 3.0 × 3.4 cm cystic lesion in the pancreatic tail; persistent main PD dilatation with apparent communication with a fluid collection superior to the pancreatic head, raising suspicion for a leak at the pancreatic neck; the presence of a percutaneous pigtail drain, with persistent loculated ascites and peritoneal thickening suggestive of peritonitis; a nonocclusive portal vein thrombus extending into the right portal vein; and a nearly occlusive arterial thrombus in the distal left common iliac artery extending into the proximal external and internal iliac arteries.

Secretin-stimulated MRCP during this admission demonstrated communication between the dominant head/uncinate pseudocyst (6.3 × 7.2 cm) and the main PD on post-secretin imaging; a tail collection (3.7 × 4.6 cm) suspected to communicate similarly; diffuse main PD dilatation with filling defects suggestive of intraductal stones; and only small-volume ascites, representing a marked improvement from prior imaging (Figure 3).

**Figure 3:**
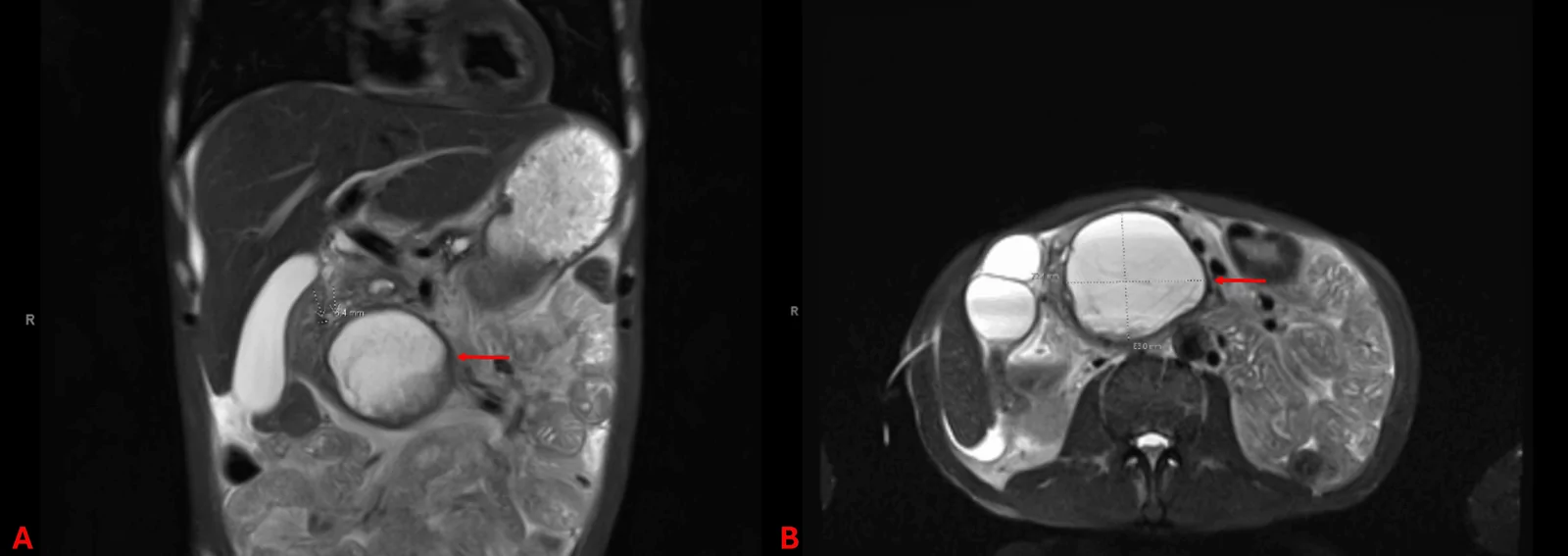
Coronal T2-weighted (Panel A) and axial T2-weighted (Panel B) MRI sequences obtained during secretin-stimulated MRCP demonstrate the dominant pancreatic pseudocyst centered in the pancreatic head and uncinate process region (red arrows in both panels). In Panel A, the pseudocyst appears as a well-circumscribed, predominantly T2-hyperintense fluid collection with internal heterogeneity that may reflect proteinaceous or hemorrhagic debris. An adjacent, unmarked, smaller peripancreatic fluid collection measuring approximately 4 mm is also visible. In Panel B, the dominant pseudocyst measures 6.3 × 7.2 cm, as illustrated by the measurement calipers, and demonstrates similar heterogeneous internal signal characteristics. On post-secretin imaging, the collection demonstrated increased fluid signal, suggesting communication with the main pancreatic duct. A separate fluid collection in the pancreatic tail region, not shown in these panels, measured 3.7 × 4.6 cm, demonstrated similar proteinaceous or hemorrhagic signal characteristics, and was also suspected to communicate with the main pancreatic duct. MRCP: Magnetic resonance cholangiopancreatography.

Transthoracic echocardiography (TTE) demonstrated an ejection fraction of 68-72%, making systolic heart failure unlikely as a contributor to the ascites. Apixaban, initiated for PVT, was held before the planned endoscopic procedures.

ERCP was attempted with the therapeutic intent of achieving transpapillary PD drainage to divert pancreatic secretions into the duodenum and reduce ongoing leakage into the peritoneum, rather than for diagnostic purposes or as source control for an established infection. The procedure was technically extremely difficult because of lateral displacement of the papilla, congested and redundant duodenal folds, duodenal spasm, and extensive calcifications in the pancreatic head. Despite multiple attempts using guidewires and sphincterotomes, neither ventral nor dorsal PD cannulation could be achieved. Biliary cannulation was successful, and a limited sphincterotomy was performed to facilitate future attempts at PD access; the bile duct was swept, and no abnormalities were identified. Acute care surgery and interventional radiology were subsequently consulted.

Percutaneous image-guided placement of a 12-French pigtail drain into the right upper-quadrant peritoneal collection yielded 3.3 L of cloudy yellow ascitic fluid, with serial drain output monitored thereafter.

The patient subsequently developed acute left lower-extremity ischemia, with pain, pallor, and pulselessness. CT confirmed a nearly occlusive left common iliac arterial thrombus extending into the proximal iliac vessels, and duplex USG confirmed absent arterial perfusion in the left lower extremity. Given its temporal association with severe intra-abdominal inflammation and active PVT, this arterial thromboembolism was clinically attributed to a pancreatitis-associated prothrombotic state. A dedicated evaluation for inherited or acquired thrombophilia, such as antiphospholipid antibody testing or, if clinically indicated, JAK2 mutation analysis, was not pursued during this admission; outpatient consideration of such testing may be warranted if a recurrent event occurs. Vascular surgery performed an emergency left aortoiliac thrombectomy through right femoral access using a Pounce thrombectomy device, followed by selective tibial angioplasty and repeat thrombectomy five days later. The clinical course was subsequently complicated by rhabdomyolysis and impending compartment syndrome, requiring four-compartment fasciotomy of the left lower extremity, with wound vacuum-assisted closure placement and subsequent wound closure. A therapeutic heparin infusion, required for the vascular indication, precluded repeat pancreatic endoscopic intervention during this admission.

The peritoneal drain subsequently yielded approximately 1,650 mL of chylous fluid, with a triglyceride level of 725 mg/dL confirming chylous ascites. A strict very-low-fat diet and total parenteral nutrition (TPN) were initiated, with octreotide added because of persistently high drain output. The acute care surgery service assumed primary management, with vascular surgery continuing to follow the patient.

Follow-up CT demonstrated near-complete resolution of the portal venous thrombus and decreased ascites, although the pseudocysts had increased slightly in size (head/uncinate, 7.8 cm; tail, 4.3 cm). A later secretin-enhanced MRCP demonstrated interval reduction of the pancreatic-head collection to approximately 26.8 × 17.4 mm, with no evidence of an active PD leak and persistent communication between the collection and the main PD (Figure 4). The advanced gastroenterology team determined that no further inpatient endoscopic intervention was warranted, and outpatient follow-up was planned for possible pseudocyst drainage and ERCP reassessment.

**Figure 4:**
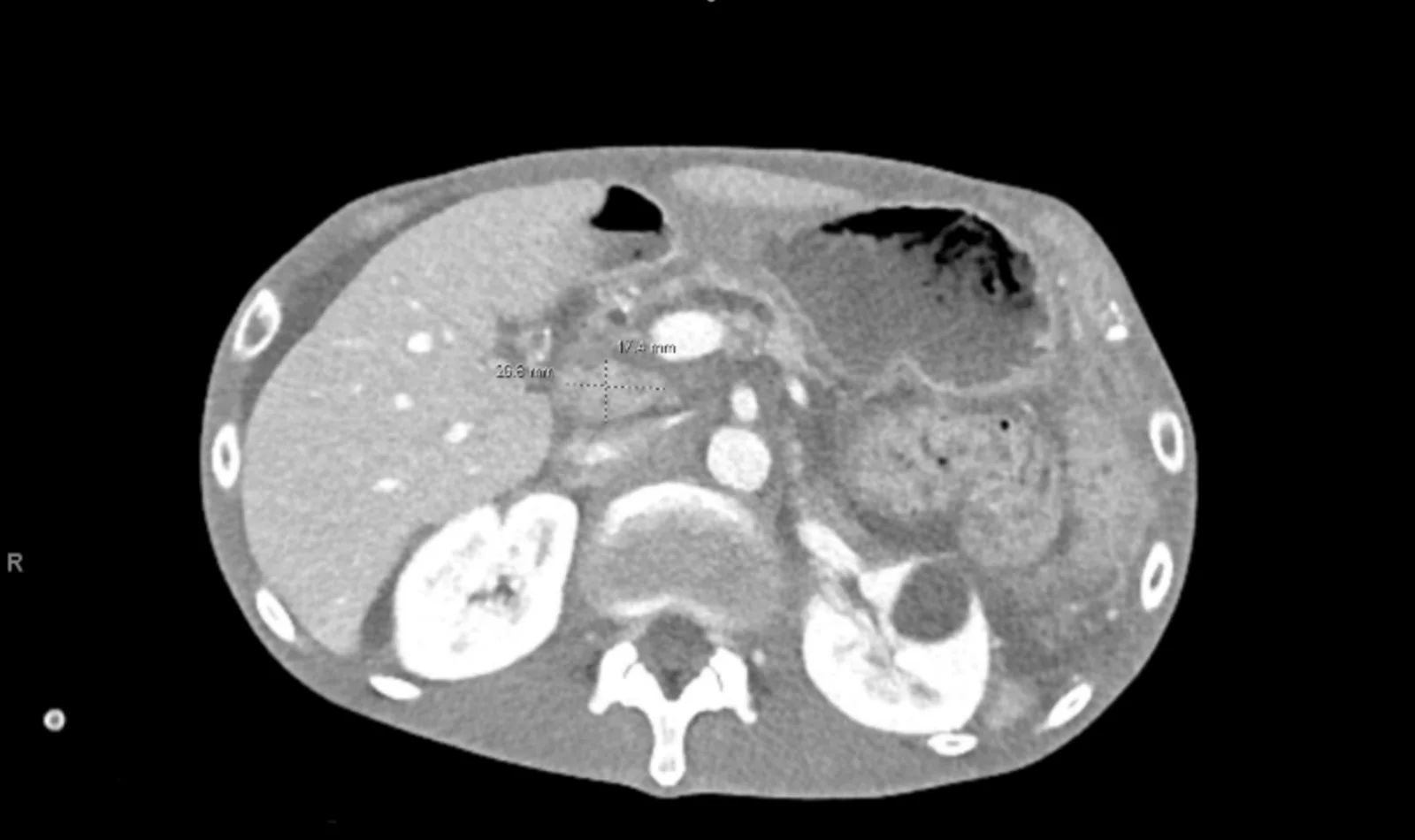
Axial T2-weighted MR image obtained during a later MRCP examination, demonstrating interval improvement following conservative management. The pancreatic-head fluid collection measures approximately 26.8 × 17.4 mm, representing a decrease from the 7.8-cm measurement reported on the preceding follow-up CT. No new pancreatic fluid collections are identified. MRCP: Magnetic resonance cholangiopancreatography.

Octreotide and TPN were discontinued as the drain output became serous and the patient tolerated oral intake without difficulty. He was discharged on a low-fat diet and a short, defined course of oral ciprofloxacin intended to bridge the peridischarge period while the ascites and drain status continued to resolve, rather than to provide indefinite, cirrhosis-style secondary prophylaxis for SBP. He was also discharged on rivaroxaban and aspirin, with close outpatient follow-up scheduled. Formal criteria for discontinuing ciprofloxacin were not established at discharge; this gap is discussed below.

The key objective findings of this case are summarized as follows. The patient, a 50-year-old non-cirrhotic man with chronic calcific pancreatitis secondary to alcohol use disorder, presented with massive pancreatic ascites from an imaging-supported PD leak suspected at the pancreatic neck. This was complicated by neutrocytic ascites (PMN leukocyte count ≥250 cells/mm³), provisionally classified as CNNA, with a clinical syndrome highly consistent with SBP. No imaging or laboratory evidence of cirrhosis was identified; liver enzyme levels, synthetic function, and bilirubin levels remained normal throughout the admission.

The ascites was multifactorial: primarily pancreatic, given the markedly elevated amylase level, with a secondary contribution from portal hypertension associated with nonocclusive PVT. The PVT was considered related to peripancreatic inflammation and SMV compression by the dominant pseudocyst. A significant cardiac contribution was considered unlikely based on TTE. Peritoneal inflammation was supported both clinically by abdominal tenderness and an elevated ascitic PMN count and radiographically by peritoneal thickening on CT. However, as discussed below, a definitive bacteriological distinction between infection and sterile chemical peritonitis could not be established because of the negative culture and incomplete data required to apply Runyon’s criteria.

Endoscopic PD drainage was unsuccessful because of difficult anatomy and the subsequent need for uninterrupted anticoagulation. Conservative management, including IV antibiotics, peritoneal drainage, TPN, a low-fat diet, and octreotide, resulted in no evidence of an active PD leak on secretin-enhanced MRCP, clinical resolution of the ascites without recurrence, and safe discharge. The arterial thrombosis and compartment syndrome were successfully managed with thrombectomy and fasciotomy, respectively; the PVT resolved nearly completely; and the chylous ascites resolved with conservative measures (Table 2).

**Table 2:** Clinical timeline from day 0 (admission) to discharge. CNNA: Culture-negative neutrocytic ascites; EF: Ejection fraction; ERCP: Endoscopic retrograde cholangiopancreatography; MRCP: Magnetic resonance cholangiopancreatography; PD: Pancreatic duct; PMN: Polymorphonuclear leukocyte; PVT: Portal vein thrombosis; SAAG: Serum-ascites albumin gradient; SBP: Spontaneous bacterial peritonitis; TPN: Total parenteral nutrition; TTE: Transthoracic echocardiography; VAC: Vacuum-assisted closure.

Timepoint	Key Events
Day 0	Presentation with progressive abdominal distention. CT demonstrates chronic calcific pancreatitis, pancreatic pseudocysts, a nonocclusive thrombus in the main and right portal veins, findings suggestive of portal enteropathy, and moderate-to-large-volume ascites.
Days 1-2	Diagnostic and therapeutic paracentesis (2.2 L) reveals an ascitic PMN count ≥250 cells/mm³, an amylase level of 14,619 U/L, and a SAAG of 1.5 g/dL. The ascitic findings are provisionally classified as CNNA, with a clinical syndrome consistent with SBP; intravenous ceftriaxone is initiated. TTE excludes cardiac cause (EF 68-72%), making a systolic cardiac cause unlikely.
Week 1	MRCP identifies a pancreatic head duct stricture, upstream ductal dilatation, and a dominant uncinate pseudocyst; no definitive leak site is visualized. Repeat CT demonstrates reaccumulation of ascites and enlargement of the pseudocyst.
Weeks 1-2	ERCP is attempted for therapeutic PD drainage; ventral and dorsal PD cannulation is unsuccessful because of calcifications and distorted anatomy. Biliary sphincterotomy is performed. Interventional radiology places a percutaneous peritoneal drain, removing 3.3 L of cloudy fluid.
Week 2	Acute left lower-extremity ischemia necessitates emergency aortoiliac thrombectomy, followed by repeat thrombectomy and angioplasty five days later. Rhabdomyolysis with impending compartment syndrome necessitates four-compartment fasciotomy with wound VAC placement.
Weeks 2-3	Drain output is identified as chylous, with a triglyceride level of 725 mg/dL. A strict very-low-fat diet, TPN, and octreotide are initiated. Therapeutic anticoagulation is maintained for the vascular indication, precluding repeat endoscopy.
Weeks 3-4	Follow-up CT demonstrates near-complete resolution of the PVT and decreased ascites. Secretin-enhanced MRCP shows no evidence of an active PD leak, although communication between the pseudocyst and the main PD persists.
Discharge	Octreotide and TPN are discontinued, and the patient tolerates oral intake while following a low-fat diet. He is discharged on a short course of oral ciprofloxacin, rivaroxaban, and aspirin, with close outpatient follow-up for pseudocyst reassessment.

## Discussion

This case illustrates the rare and diagnostically challenging occurrence of neutrocytic ascites clinically consistent with SBP in a non-cirrhotic patient whose ascites was primarily pancreatic in origin and driven by a PD leak. Classical teaching situates SBP firmly within liver cirrhosis; non-cirrhotic SBP is far less familiar and, as this case demonstrates, may be difficult to distinguish confidently from CNNA, infected pancreatic fluid collections, and sterile chemical peritonitis.

The pathophysiology of SBP in cirrhotic patients is well established and involves altered gut microbiota and permeability, impaired hepatic Kupffer cell function, reduced ascitic complement and opsonin activity, and bacterial translocation through the mesenteric lymph nodes [2,10]. Our patient did not have cirrhosis or the hepatic dysfunction that typically underlies these derangements; therefore, the mechanism of any bacterial inoculation must be understood differently. We hypothesize that massive pancreatic ascites from a PD leak, creating a large, enzyme-rich peritoneal compartment with an altered protein composition, combined with localized portal hypertension from PVT, may have facilitated bacterial translocation. The PVT was itself considered to have been provoked by peripancreatic inflammation and SMV compression by the pseudocyst. PVT may promote intestinal venous congestion, bowel wall edema, as suggested by small-bowel thickening on imaging, and increased permeability, potentially permitting bacterial translocation even in the absence of hepatic parenchymal disease [7,11].

An elevated SAAG (≥1.1 g/dL; 1.5 g/dL in this case) is generally interpreted as a marker of portal hypertension; this case illustrates how SAAG elevation can arise from non-hepatic causes such as PVT. A SAAG ≥1.1 g/dL in a non-cirrhotic patient should prompt evaluation for extrahepatic portal hypertension, including splenic or PVT, rather than being assumed to indicate cirrhosis [5,12]. The markedly elevated ascitic amylase level (14,619 U/L) correctly redirected the diagnostic evaluation toward a pancreatic etiology. We recommend considering ascitic amylase measurement when evaluating non-cirrhotic ascites of uncertain cause, particularly in patients with suspected pancreatic disease.

Distinguishing true bacterial infection from sterile chemical peritonitis in pancreatic ascites is genuinely difficult, and we wish to be explicit about the limits of our diagnostic certainty in this case. The PMN leukocyte threshold of ≥250 cells/mm³ was validated primarily in cirrhotic ascites. Pancreatic enzymes entering the peritoneum can independently elevate PMN counts through chemical irritation. Furthermore, although the PD is anatomically continuous with the duodenum via the ampulla of Vater, whether this connection permits microbial entry in the setting of a PD leak remains uncertain. Thus, a PD leak may not create the same type of “closed,” gut-isolated compartment presumed to exist in uncomplicated cirrhotic ascites. Because the ascitic fluid culture was negative and glucose, LDH, and total protein levels were not obtained during the initial paracentesis, precluding formal application of Runyon’s criteria, we cannot definitively exclude secondary bacterial peritonitis or determine whether the neutrophilia reflected infection, chemical irritation, or both. Per established guidelines, empiric antibiotic therapy is nonetheless warranted once the PMN threshold is met, irrespective of culture positivity [1,13], which is why ceftriaxone was appropriately initiated pending the diagnostic workup described above. Therefore, ceftriaxone was appropriately initiated while the diagnostic evaluation described above was pursued. We provisionally classify this case as CNNA with a clinical syndrome highly consistent with SBP rather than asserting culture-confirmed SBP outright. We believe that this more cautious terminology is itself an important message of the report: clinicians should recognize both the possibility of bacterial translocation in this setting and the genuine diagnostic ambiguity that pancreatic ascites introduces into the standard SBP criteria.

Regarding management of the PD leak, ERCP with transpapillary PD stenting is the preferred endoscopic intervention when intervention is required, aiming to redirect pancreatic secretions internally and seal the leak [14]. In this patient, ERCP was pursued specifically as a therapeutic attempt at ductal drainage rather than as source control for a proven infection. However, successful ductal drainage could also have indirectly reduced the risk of infection by decreasing the volume of enzyme-rich fluid potentially susceptible to infection. This potential dual role is one reason we hesitate to describe the peritonitis as “spontaneous” in the strictest sense and further supports our use of CNNA/SBP-consistent terminology. Extensive calcifications, a distorted and laterally displaced papilla, and redundant duodenal folds rendered PD cannulation technically impossible despite expert attempts, underscoring a well-described challenge in the endoscopic management of advanced chronic calcific pancreatitis. Alternative approaches, including endoscopic ultrasound (EUS)-guided cystogastrostomy or cystoduodenostomy, EUS-guided rendezvous, and surgical drainage using Frey or Puestow procedures, remain options in refractory cases [14,15]. In our patient, the concurrent need for uninterrupted anticoagulation for acute aortoiliac thromboembolism precluded repeat endoscopic attempts during hospitalization. Spontaneous resolution of the PD leak, demonstrated by the absence of an active leak on secretin-enhanced MRCP, represents an uncommonly favorable outcome reported in a minority of conservatively managed cases [16].

The concurrent aortoiliac thromboembolism was considered likely to reflect a prothrombotic state associated with systemic inflammation, immobility, and the hypercoagulable milieu of chronic pancreatitis, particularly in the setting of concurrent active PVT. As noted above, a dedicated thrombophilia workup was not performed during this admission. We consider an inflammatory or pancreatitis-associated mechanism the most parsimonious explanation given the clinical context, although a contributing hereditary or acquired thrombophilic disorder cannot be fully excluded. This vascular complication substantially prolonged the hospitalization and illustrates the multisystem nature of advanced alcohol-related pancreatitis, reinforcing the importance of clinical vigilance for thromboembolic complications in these patients.

Chylous ascites, confirmed by a drain fluid triglyceride level of 725 mg/dL, represents an additional rare complication, likely resulting from disruption of the mesenteric lymphatic channels by peripancreatic inflammation or lymphatic obstruction caused by adjacent pseudocysts and reactive lymphadenopathy [17]. Management using a strict low-fat diet, TPN, and octreotide is consistent with standard conservative approaches and was successful in this case.

The rationale for antibiotic prophylaxis at discharge merits explicit discussion. Secondary SBP prophylaxis is a well-established, evidence-based recommendation for cirrhotic patients with a previous episode of SBP, persistent ascites, and an ongoing risk of recurrence; however, this evidence base does not directly apply to a non-cirrhotic patient whose ascites was pancreatic in origin and whose PD leak had resolved. Ciprofloxacin was intended as a short, time-limited course bridging the peridischarge period while the ascites and drain status continued to resolve, rather than as indefinite secondary prophylaxis. However, formal discontinuation criteria were not prespecified at discharge, and we identify this as a gap. Future management of analogous cases would benefit from predefined discontinuation criteria, such as discontinuation once the ascites and drain output have resolved, together with a defined outpatient follow-up interval, rather than open-ended prophylaxis extrapolated from the cirrhosis literature.

Comparison with the limited existing literature on non-cirrhotic SBP is instructive. Previous reports of non-cirrhotic neutrocytic ascites or SBP-like syndromes have most often involved nephrotic syndrome, malignant ascites, or cardiac ascites, in which the mechanisms of translocation or infection differ substantially from those proposed here [5]. To our knowledge, this may be the first comprehensively reported case combining an imaging-supported PD leak, secretin-enhanced MRCP evidence of spontaneous ductal healing, and concurrent PVT-associated portal hypertension in a patient with preserved hepatic synthetic function. This represents a proposed distinct mechanism, PD leak-associated, PVT-facilitated bacterial translocation, that we believe warrants recognition separately from these other non-cirrhotic etiologies.

This case carries several generalizable clinical lessons. Neutrocytic ascites clinically consistent with SBP is not exclusive to cirrhotic patients; conditions producing large-volume ascites, including pancreatic ascites from a PD leak, may present with this syndrome. Ascitic fluid amylase should be considered as part of the diagnostic evaluation of unexplained ascites in a non-cirrhotic patient, particularly when pancreatic disease is suspected. An elevated SAAG in a non-cirrhotic patient should prompt evaluation for extrahepatic portal hypertension rather than an assumption of cirrhosis. Where possible, ascitic fluid evaluation should include the tests required to apply Runyon’s criteria, glucose, LDH, and total protein, along with bedside inoculation into blood culture bottles to improve the diagnostic yield when distinguishing SBP/CNNA from secondary bacterial peritonitis and sterile chemical peritonitis. Finally, early multidisciplinary involvement from advanced endoscopy, interventional radiology, acute care surgery, and vascular surgery may be essential for achieving optimal outcomes in the complex, multisystem presentations that characterize advanced alcohol-related pancreatitis.

This report is limited by its single-case and retrospective nature, which precludes the generalization of outcome data. Ascitic fluid was not inoculated into blood culture bottles at the bedside, which may have reduced the sensitivity for detecting true bacterial infection. The complete ascitic fluid tests required to apply Runyon’s criteria, glucose, LDH, and total protein, were not obtained during the initial paracentesis, preventing their formal application to assess for secondary bacterial peritonitis. No repeat paracentesis was performed to confirm ascitic PMN clearance after antibiotic initiation. A dedicated thrombophilia workup was not pursued despite the severe concurrent arterial thromboembolism. Given these limitations, the diagnosis in this case is best understood as CNNA with a syndrome highly consistent with, but not bacteriologically confirmed as, SBP. Long-term follow-up data regarding recurrence, pseudocyst evolution, response to ongoing anticoagulation for PVT, and outcomes after discontinuation of prophylactic antibiotics are not yet available. Future case series specifically examining the bacteriology, optimal diagnostic criteria, and outcomes of SBP/CNNA in non-cirrhotic pancreatic ascites would substantially add to the evidence base.

## Conclusions

This case underscores that neutrocytic ascites consistent with SBP can occur in non-cirrhotic patients when a PD leak and localized portal hypertension coexist, potentially creating an environment permissive to bacterial translocation. However, such cases often cannot be bacteriologically confirmed as SBP in the strict sense because of negative cultures, incomplete data required to apply Runyon’s criteria, and the anatomical continuity of the PD with the gastrointestinal lumen. We therefore favor the more precise designation of CNNA with an SBP-consistent clinical syndrome in analogous cases rather than an unqualified diagnosis of SBP.

Several practical lessons emerge for clinicians managing complex non-cirrhotic ascites: measure ascitic fluid amylase early when the etiology is unclear; investigate extrahepatic causes of portal hypertension, such as portal vein thrombosis, when the SAAG is elevated in the absence of cirrhosis; obtain the complete ascitic fluid tests required to apply Runyon’s criteria and, where feasible, inoculate ascitic fluid into blood culture bottles at the bedside to improve diagnostic certainty; and involve advanced endoscopy, interventional radiology, and relevant surgical subspecialties early, given the potential for rapid multisystem decompensation. In this patient, conservative management, including intravenous antibiotics, peritoneal drainage, total parenteral nutrition, a low-fat diet, and octreotide, resulted in spontaneous resolution of the PD leak and clinical resolution of the ascites without recurrence despite unsuccessful endoscopic intervention. This outcome demonstrates that conservative management may be effective in selected, technically challenging cases that are not amenable to endoscopic intervention.

## References

[1] Runyon BA (2009). Management of adult patients with ascites due to cirrhosis: an update. Hepatology.

[2] Wiest R, Garcia-Tsao G (2005). Bacterial translocation (BT) in cirrhosis. Hepatology.

[3] Li YT, Huang JR, Peng ML (2020). Current status and prospects of spontaneous peritonitis in patients with cirrhosis. Biomed Res Int.

[4] Tandon P, Garcia-Tsao G (2011). Renal dysfunction is the most important independent predictor of mortality in cirrhotic patients with spontaneous bacterial peritonitis. Clin Gastroenterol Hepatol.

[5] Such J, Runyon BA (1998). Spontaneous bacterial peritonitis. Clin Infect Dis.

[6] Gupta R, Patel K, Calder PC, Yaqoob P, Primrose JN, Johnson CD (2003). A randomised clinical trial to assess the effect of total enteral and total parenteral nutritional support on metabolic, inflammatory and oxidative markers in patients with predicted severe acute pancreatitis (APACHE II > or =6). Pancreatology.

[7] Chaudhry H, Sohal A, Bains K (2023). Incidence and factors associated with portal vein thrombosis in patients with acute pancreatitis: a United States national retrospective study. Pancreatology.

[8] Garg PK, Sharma M, Madan K, Saluja A, Bhardwaj R, Bhardwaj R (2011). Primary conservative treatment results in successful outcomes in patients with pancreatic ascites and pleural effusion. Am J Gastroenterol.

[9] Koulaouzidis A, Bhat S, Karagiannidis A, Tan WC, Linaker BD (2007). Spontaneous bacterial peritonitis. Postgrad Med J.

[10] Piano S, Bunchorntavakul C, Marciano S, Rajender RK (2024). Infections in cirrhosis. Lancet Gastroenterol Hepatol.

[11] Ponziani FR, Zocco MA, Campanale C (2010). Portal vein thrombosis: insight into physiopathology, diagnosis, and treatment. World J Gastroenterol.

[12] Spaander MC, van Buuren HR, Hansen BE, Janssen HL (2010). Ascites in patients with noncirrhotic nonmalignant extrahepatic portal vein thrombosis. Aliment Pharmacol Ther.

[13] European Association for the Study of the Liver (2010). EASL clinical practice guidelines on the management of ascites, spontaneous bacterial peritonitis, and hepatorenal syndrome in cirrhosis. J Hepatol.

[14] Varadarajulu S, Noone TC, Tutuian R, Hawes RH, Cotton PB (2005). Predictors of outcome in pancreatic duct disruption managed by endoscopic transpapillary stent placement. Gastrointest Endosc.

[15] Strand DS, Law RJ, Yang D, Elmunzer BJ (2022). AGA clinical practice update on the endoscopic approach to recurrent acute and chronic pancreatitis: expert review. Gastroenterology.

[16] Norman M, Moldovan S, Seghers V, Wang XP, DeMayo FJ, Brunicardi FC (2002). Sulfonylurea receptor knockout causes glucose intolerance in mice that is not alleviated by concomitant somatostatin subtype receptor 5 knockout. Ann Surg.

[17] Lizaola B, Bonder A, Trivedi HD, Tapper EB, Cardenas A (2017). Review article: the diagnostic approach and current management of chylous ascites. Aliment Pharmacol Ther.

